# The visual system of harvestmen (Opiliones, Arachnida, Chelicerata) – a re-examination

**DOI:** 10.1186/s12983-016-0182-9

**Published:** 2016-11-16

**Authors:** Tobias Lehmann, Eva Lodde-Bensch, Roland R. Melzer, Martina Metz

**Affiliations:** 1 Bavarian State Collection of Zoology, SNSB, Münchhausenstraße 21, 81247 Munich, Germany; 2 Department Biologie II, Ludwig-Maximilians-Universität München, Großhaderner Straße 2, 82152 Planegg-Martinsried, Germany; 3 GeoBioCenter, LMU, Richard -Wagner-Str. 10, 80333 Munich, Germany

**Keywords:** Chelicerata, Arachnida, Opiliones, Visual system, Central projections, Phylogeny

## Abstract

**Background:**

The visual systems in chelicerates are poorly understood, even though they show strong variation in eye and visual neuropil architecture, thus may provide valuable insights for the understanding of chelicerate phylogeny and eye evolution. Comparable morphological characters are desperately sought for reconstructions of the phylogeny of Chelicerata, especially with respect to Arachnida. So far, reliable data exist only for Pycnogonida, Xiphosura, Scorpiones, and Araneae. The few earlier studies of the organisation of the visual system in harvestmen are contradictory concerning the number, morphology, and position of the visual neuropils.

**Results:**

We undertook a descriptive and comparative analysis of the neuroanatomy of the visual system in several phalangid harvestmen species. Various traditional and modern methods were used that allow comparisons with previous results (cobalt fills, DiI/DiO labelling, osmium ethyl gallate procedure, and TEM). The R-cells (photoreceptor and arhabdomeric cells) in the eyes of Opiliones are linked to a first and a second visual neuropil. The first visual neuropil receives input from all R-cell axons, in the second only few R-cells terminate in the distal part. Hence, the second visual neuropil is subdivided in a part with direct R-cell input and a part without. The arcuate body is located in a subsequent position with direct contact to the second visual neuropil.

**Conclusions:**

This re-examination comes to conclusions different from those of all previous studies. The visual system of phalangid Opiliones occupies an intermediate position between Pycnogonida, Xiphosura, and Scorpiones on the one side, and Araneae on the other side. The projection of the R-cells is similar to that in the former grouping, the general neuropil arrangement to that in the latter taxon. However, more research on the visual systems in other chelicerate orders is needed in order to draw inferences on phylogeny or eye evolution.

## Background

According to recent theories about the phylogeny of Opiliones (harvestmen) there are two main lineages, Cyphophthalmi as the basal suborder, and Phalangida as its sister group comprising all other harvestmen, but their position within Arachnida remains unsolved [1–5]. Many, but not all, phalangid harvestmen possess a pair of everse median eyes with a cuticular lens on a dorsomedian eye tubercle or ocularium situated on the prosoma. In some representatives of, e.g., Stygnommatidae, Biantidae and Dibuninae, an eye tubercle is absent and the eyes are located in more lateral positions. Moreover, eyes on a median eye tubercle are not present in Cyphophthalmi. Many cyphophthalmids are eyeless, but some representatives of this lineage (Pettalidae and Stylocellidae) have laterally positioned eyes [6, 7]. Ultrastructurally these eyes are interpreted as laterally displaced median eyes [8]. Recently a fossil harvestman was also described with four eyes, interpreted as two median and two lateral eyes as in, e.g., Xiphosura and Scorpiones [1]. In the same study, gene expression in the extant species *Phalangium opilio* demonstrated vestiges of lateral eye tubercles. This, in turn, would mean that the presence of both median and lateral eye types is a plesiomorphic state lost in recent Opiliones. Furthermore, the cyphophthalmid eyes could be true arachnid lateral eyes.

Thus, it is not unequivocally clear whether the 'median eye' term often applied to harvestmen eyes is merely topological or also informative in an evolutionary context as it is in many other arthropods, in which a distinction of median and lateral eyes and neuropils with respect to position, structure and function is evident (see Lehmann et al. [9] for a recent review of chelicerate visual systems). In Opiliones, a taxon with only one of the two eye classes present, this question is not trivial, which was one of the motivations for the present study.

The eye of a phalangid harvestman is composed of a dioptric apparatus comprising a biconvex lens and a crystalline body made by lentigene cells, and of a preretinal membrane. The proximal part of the eye contains photoreceptor cells, arhabdomeric cells and glia cells in a distinct arrangement: the R-cells (or retinula cells, i.e. photoreceptor and arhabdomeric cells) form units of three to four photoreceptor cells and their rhabdomeres, each associated with an arhabdomeric cell [10, 11]. The arhabdomeric cells are seen as non-photosensitive, secondary neurons, and are found in similar form in Xiphosura and Scorpiones [10, 12].

In a typical harvestman, the visual field of the two eyes on the eye tubercle extends laterally, and it has been suggested that in many species the eyes provide a quite rough image of light and dark structures rather than a sharp image [13]. Several laboratory experiments have reported that species of Phalangida show negative phototaxis. [13]. Many harvestmen are active during the night, and feed on carrion, fungi or dead organic material rather than being carnivorous. High resolution vision therefore is not necessary in these species. Meyer-Rochow & Liddle [11] showed that two cave inhabiting harvestmen species feeding on glow-worms (*Arachnocampa lumin*osa) are positively phototactic for small light sources. The harvestmen studied in the present analyses are at least partly active during the day. In a species of *Leiobunum*, Willemart et al. [13] observed that a large, dark object provoked escape behaviour.

For various chelicerate taxa knowledge on the neuropils processing the visual input is cursory and insufficient for comparative analyses across Chelicerata to understand the evolution of their visual systems and include the character sets in a neurophylogenetical context. However, this approach has proven fruitful in recent comparative analyses of the visual systems of Pycnogonida and Scorpiones [9, 14–16]. Concerning other chelicerate taxa, recent data exist only for the xiphosuran, *Limulus polyphemus* [17–20], an important species well investigated in the field of visual neuroscience, and for Araneae [21–24].

The visual neuropils of Opiliones have been analysed in a few studies in the past, but the results are partly unclear and contradictory with respect to the position and number of visual neuropils, presence or absence of chiasmata, and the projection patterns of visual fibres. The first of these studies – without any doubt an arthropod neuroanatomy classic – was the one by Saint Remy [25], followed by Holmgren [26] and Hanström [27–29]. The only detailed modern analysis is the one by Breidbach & Wegerhoff [30], but this study did not manage to resolve the partially contradictory views in a convincing way.

In the present work we analyse the trajectories of axon bundles from the eyes to the visual neuropils, study the number, form, connectivity and general morphology of the visual neuropils, and locate the target neuropils of the axon terminals. We use various neuroanatomical techniques (Cobalt fills, DiI/DiO labelling, the Osmium ethyl gallate procedure, TEM, and AMIRA 3D-reconstruction) to examine four different species of phalangid harvestmen: *Leiobunum spec*. (Sclerosomatidae), *Opilio canestrinii* (Thorell, 1876) (Phalangiidae), *Platybunus pinetorum* (C. L. Koch, 1839) (Phalangiidae), and *Rilaena triangularis* (Herbst, 1799) (Phalangiidae).

## Results

### General layout of the visual system (Figs. 1, 2, 3, 4, 5)

**Fig. 1:**
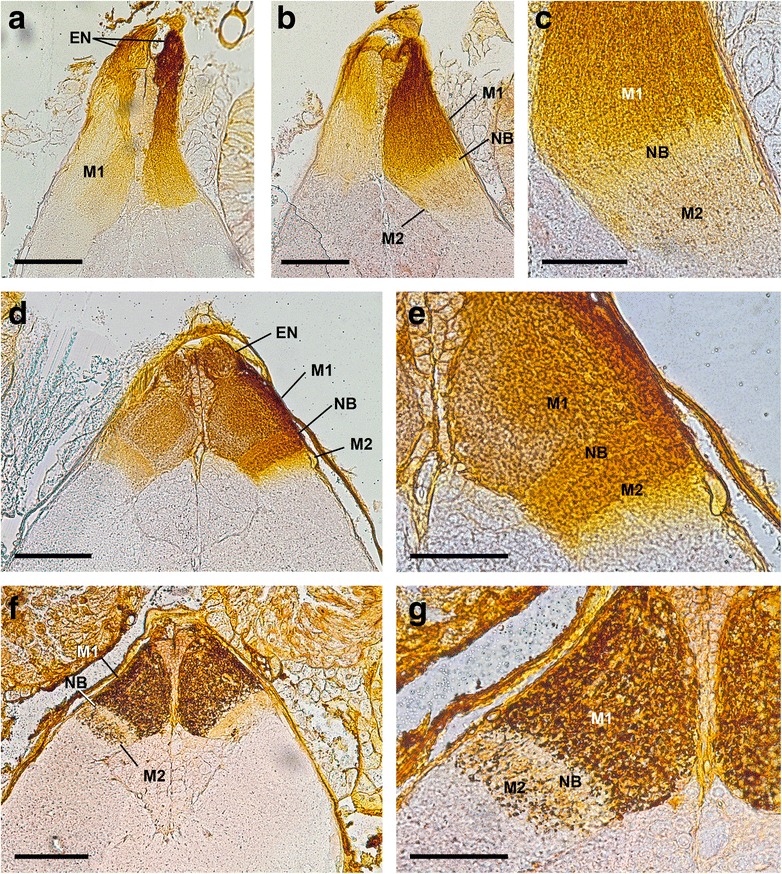
Cobalt fills via both eyes of *Leiobunum spec.* (a-c) and *Opilio canestrinii* (d-g), transversal sections, dorsal is up. **a** right eye nerve and right first visual neuropil densely filled with cobalt, left nerve and neuropil less filled. Bar 100 μm. **b** three sections after A, in right hemisphere Cobalt-filled retinula axons terminate in first visual neuropil and in dorsal part of second visual neuropil, in second neuropil fewer fibres filled. Bar 100 μm. **c** detail of right hemisphere in B with border between both neuropils and Cobalt-filled retinula axons in dorsal part of second neuropil. Bar 50 μm. **d** Cobalt-filled retinula axons terminating via eye nerve in first and second visual neuropil. Bar 100 μm. **e** detail of right hemisphere in C with border between both neuropils and Cobalt-filled retinula axons in dorsal part of second neuropil. Bar 50 μm. **f** Cobalt-filled retinula axons with varicosities terminating in first and second visual neuropil. Bar 100 μm. **g** detail of left hemisphere in F with border between both neuropils and Cobalt-filled retinula axons with varicosities in dorsal part of second neuropil. Bar 50 μm. EN, eye nerve; M, median eye visual neuropil; NB, neuropil border

**Fig. 2:**
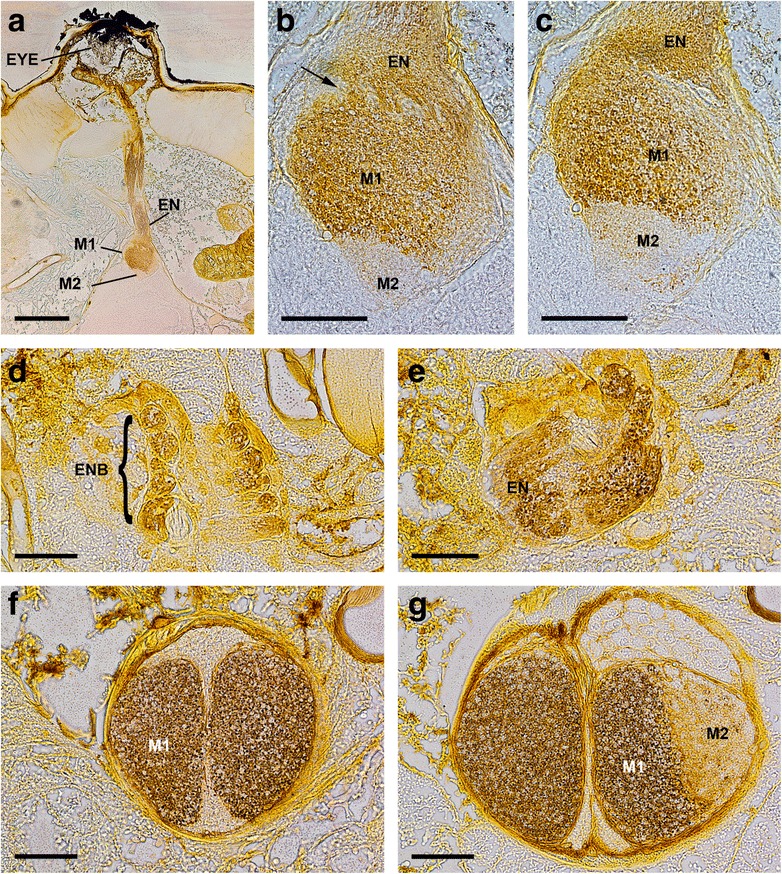
Cobalt fills via both eyes of *Opilio canestrinii* (a-c, sagittal sections, dorsal is up) and *Leiobunum spec.* (d-g, frontal sections, anterior is up). **a** eye tubercle with eye and Cobalt-filled eye nerve, first and second visual neuropil. Bar 200 μm. **b** detail of first and second visual neuropil in A, second neuropil with fewer Cobalt-filled axons. Note eye nerve separating in several bundles after entering brain (arrow). Bar 50 μm. **c** one section after B, first and second visual neuropil with Cobalt-filled retinula axons, second neuropil with fewer Cobalt-filled axons. Bar 50 μm. **d** several Cobalt-filled eye nerve bundles projecting from eye to brain. Bar 50 μm. **e** five sections after D, eye nerve bundles fuse to one eye nerve. Bar 50 μm. **f** first visual neuropils packed with Cobalt-filled retinula axons. Bar 50 μm. **g** five sections after F, in right hemisphere first visual neuropil packed with Cobalt-filled retinula axons and second visual neuropil with few Cobalt-filled retinula axons. Bar 50 μm. EN, eye nerve; ENB, eye nerve bundles; EYE, eye; M, median eye visual neuropil; NB, neuropil border

**Fig. 3:**
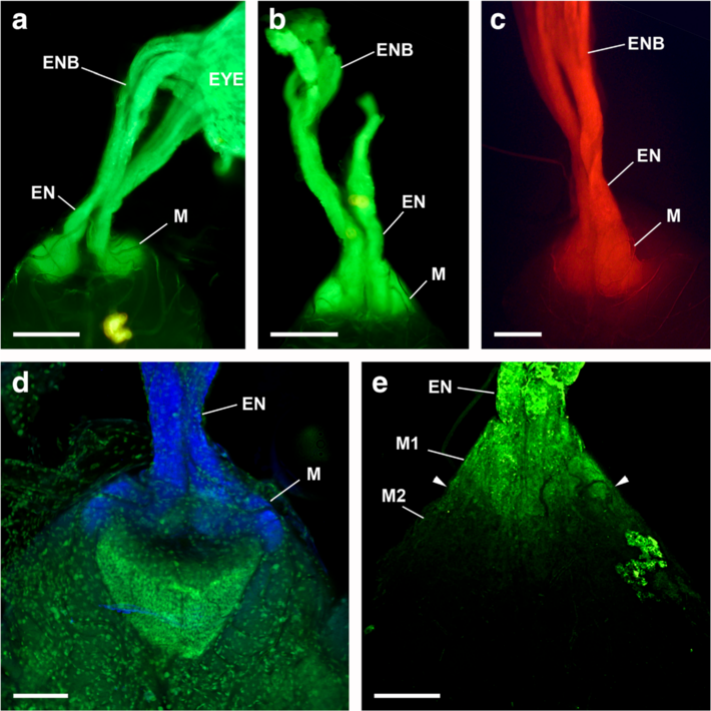
DiO (a, b, d, and e) and DiI (c) labelling via both eyes of *Opilio canestrinii*. (a-c, fluorescence microscope; d, e, CLSM; dorsal is up). **a** DiO labelled eye, eye nerve bundles, eye nerve, and visual neuropils; no distinction between first and second neuropil possible. Bar 200 μm. **b** DiO labelled eye nerve bundles, eye nerve, and visual neuropils; no distinction between first and second neuropil possible. Bar 200 μm. **c** DiI labelled eye nerve bundles, eye nerve, and visual neuropils; no distinction between first and second neuropil possible. Bar 200 μm. **d** DiO labelled eye nerve, and visual neuropils; no distinction between first and second neuropil possible. DAPI labelled cell bodies in green. Same specimen as in A. Bar 100 μm. **e** DiO labelled eye nerve, first, and second visual neuropil. Neuropil border between first and second neuropil visible (arrowheads), in second visual neuropil fewer DiO. Same specimen as in B. Bar 100 μm. EN, eye nerve; ENB, eye nerve bundles; EYE, eye; M, median eye visual neuropil

**Fig. 4:**
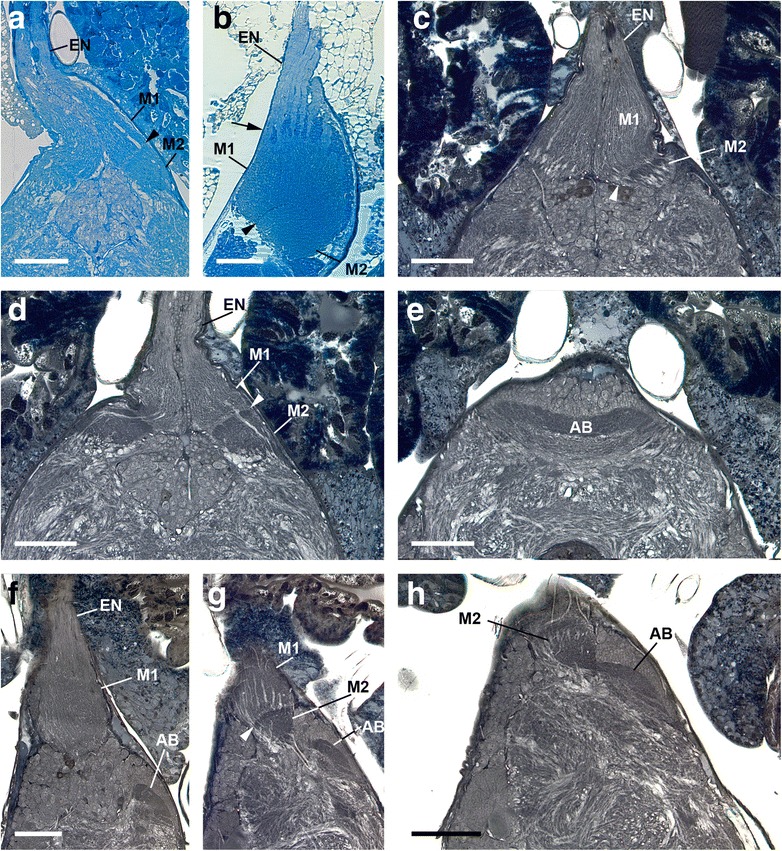
General anatomy of protocerebrum and visual neuropils (Richardson (*Platybunus pinetorum*) and Wigglesworth stains (*Leiobunum spec.*), dorsal is up). **a** transversal section showing eye nerve, first and second visual neuropil with border in between (arrowhead). Bar 100 μm. **b** sagittal section showing eye nerve, first and second visual neuropil with border in between (arrowhead); note eye nerve separating in several bundles after entering brain (arrow). Bar 100 μm. **c** transversal section with eye nerve, first visual neuropil and on right hemisphere beginning of second visual neuropil; note second neuropil darker stained. Bar 100 μm. **d** seven sections after C; first and second visual neuropil with border in between (arrowhead). Bar 100 μm. **e** 16 sections after D; arcuate body in dorso-posterior position; note arcuate body darker stained. Bar 100 μm. **f** sagittal section with first visual neuropil in anterodorsal position and arcuate body in dorso-posterior position; note arcuate body darker stained. Bar 100 μm. **g** four sections after F; first and second visual neuropil in anterodorsal position with border in between (arrowhead) and arcuate body in dorso-posterior position in close vicinity to second visual neuropil; note second visual neuropil and arcuate body darker stained. Bar 100 μm. **h** three sections after G; second visual neuropil and arcuate body contact each other. Bar 100 μm. AB, arcuate body; EN, eye nerve; M, median eye visual neuropil

**Fig. 5:**
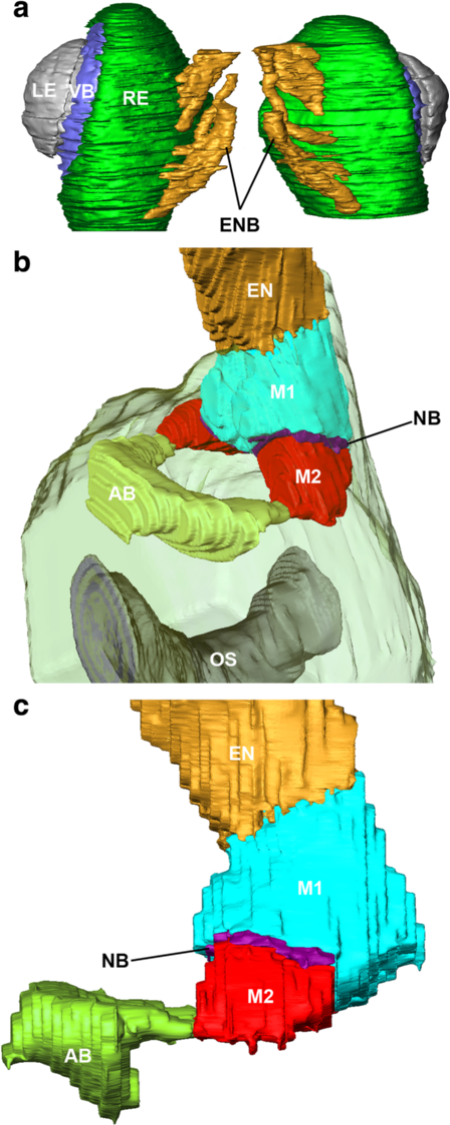
3D serial reconstruction of eye and visual neuropils. Eye reconstructed on basis of semithin sections of *Rilaena triangularis* and visual neuropils on basis of Wigglesworth stains of *Leiobunum spec.*. **a** eye composed of lens (grey), vitreous body (dark blue), and retina (dark green); note several nerve bundles (orange) exit the eye and project to protocerebrum with retained relative positions representing subsets of retinula cells. **b**, **c** dorso-lateral and lateral view showing arrangement of neuropils; orange, eye nerve; light blue, first visual neuropil; purple, neuropil border; red, second visual neuropil; light green, arcuate body. AB, arcuate body; EN, eye nerve; ENB, eye nerve bundles; LE, lens; M, median eye visual neuropil; NB, neuropil border; OE, oesophagus; RE, retina; VB, vitreous body

All the species studied here have a pair of well-developed eyes located on an eye tubercle anterodorsally on the body (Fig. 2a). In the proximal region of the eyecups several nerve bundles originate (Figs. 5a). These bundles join successively, and finally combine in a single eye nerve per hemisphere just before the nerves enter the protocerebrum (Figs. 2d, e; 3a, c). In a dorsal, tapered protrusion of the brain, the nerves project directly into the visual neuropils of the protocerebrum (Figs. 1a, c; 2a–c; 3a–e; 4a–e, f; 5b, c).

Each eye supplies two distinct, successive visual neuropils as targets of the R-cell axons (Figs. 1–5). The first visual neuropil is located in the anterodorsal tip of the protocerebrum (Figs. 1a–g; 2a–c, f, g; 3e; 4a–d, f, g; 5b, c). The right and left neuropils contact each other laterally, but without exchanging fibres. The second visual neuropil is located ventrolaterally below to the first neuropil (Figs. 1b–g; 2a–c, g; 3e; 4a–d, g, h; 5b, c). The first and second neuropils merge into each other, but with a neuropil border visible. The right and left second visual neuropils do not contact each other laterally. Furthermore, the arcuate body occupies a superficial, dorsoposterior position in the brain (Figs. 4e–h; 5b, c). Its shape is slightly bent anteriorly. Laterally the arcuate body is with direct contact to the second visual neuropils.

The visual neuropils are unequivocally identified with Cobalt fills and DiI/DiO labelling, and can also be recognised with osmium-ethyl-gallate staining, as dark-stained areas, as is typical for dense neuropils such as sensory neuropils (Figs. 1, 2, 3, 4). The arcuate body can be recognised with osmium-ethyl-gallate staining (Fig. 4).

### Eyes and eye nerve bundles

The eye is composed of a lens, a vitreous body, and the retina (Fig. 5a). In the proximal region of each eye a group of several nerve bundles, each representing a section of the retina, originates and projects ventrally to the brain. The starting points of the bundles are arranged in a row on the inner surface of the eyes (Fig. 5a). Hence, initially the eye nerve is composed of separate bundles, ensheathed as is typical for nerves.

### Eye nerve: ‘Plaited’ area and entrance into the brain

**Fig. 6:**
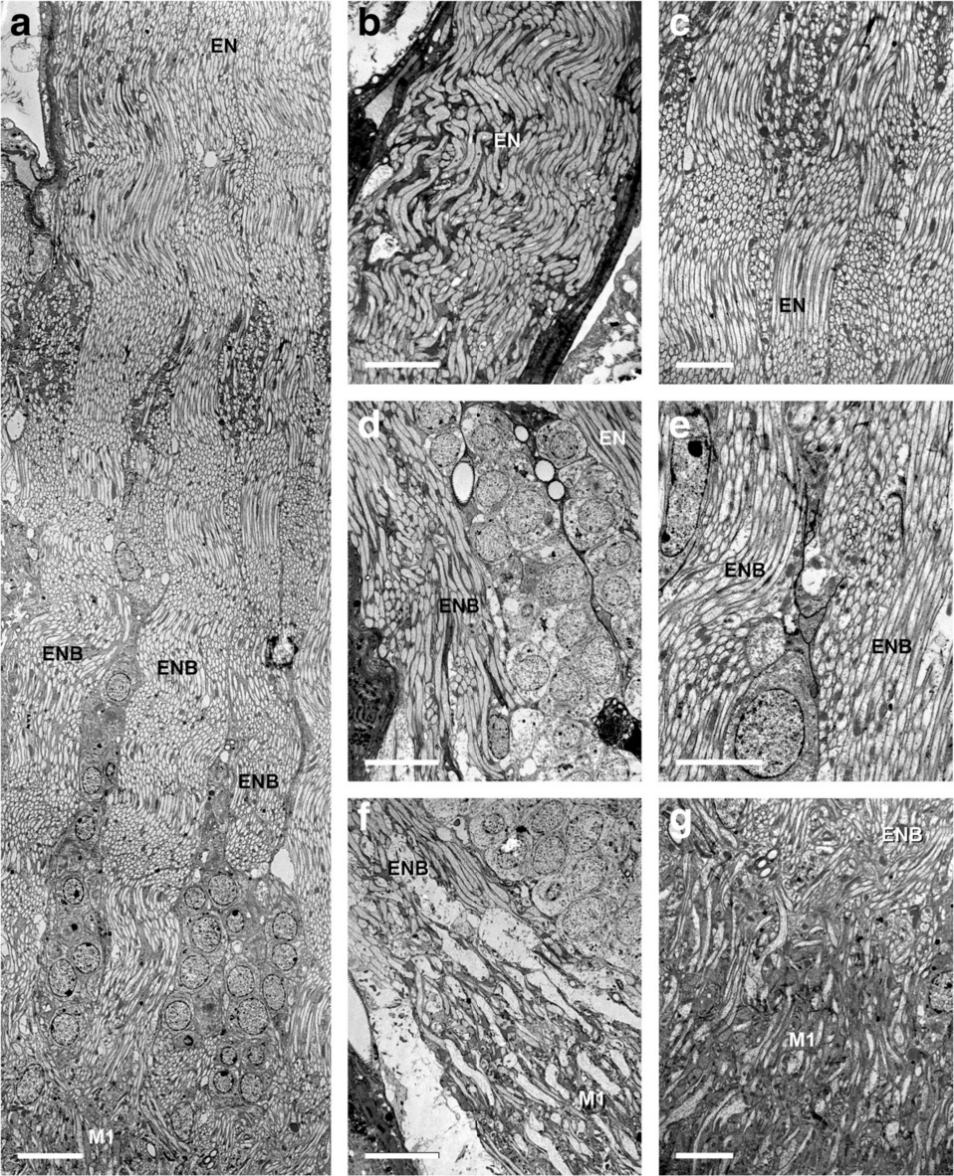
Transmission electron microscopy of *Platybunus pinetorum* of region where eye nerve enters brain and first visual neuropil (a, c, e, and g sagittal sections; b, d, f, transversal sections; dorsal is up). **a** eye nerve separates into several bundles after entering brain; with cell bodies between bundles (stitched image series). Bar 10 μm. **b**, **c** detail of eye nerve before entering the brain; note pattern of arrangement of retinula axons. Bars 10 μm and 5 μm, respectively. **d**, **e** detail of region where eye nerve separates into several nerve bundles with cell bodies in between; note no synapses in this region. Bars 10 μm and 5 μm, respectively. **e**, **f** detail of region where eye nerve bundles enter first visual neuropil. Bars 10 μm and 5 μm, respectively. EN, eye nerve; ENB, eye nerve bundles; M, median eye visual neuropil

Just distal to the tapered entrance area for the eye nerve into the brain, the bundles join and form a single nerve per hemisphere composed of densely packed axons (Figs. 2d, e; 3a, c). This was observed with the staining methods (both CoCl_2_ and DiI/DiO), Osmium ethyl gallate procedure, and TEM. With the electron microscope we observed from two different angles (transversal and sagittal) in this area, groups of axons interweaving with their neighbours, giving the nerve in this area a ‘plaited‘ appearance (Fig. 6a–c). Though we very clearly saw this redirection of axon bundles, this is restricted to small areas inside each visual nerve. Genuine chiasmatic fibres switching between the two nerves or between the extremities of the cross sections of each nerve were not observed. Moreover with TEM we exclusively found axons in this zone, dendrites of interneurons and/or synaptic connections were absent. Hence, this zone is a nerve and not a neuropil (Fig. 6a–c).

In some preparations proximal to this ‘plaited‘ area, we observed a more or less well visible annulus (e.g. Fig. 1d). This is most probably just an artefact, where the nerve is bent due to the preparation. In most other preparations no annulus is seen (e.g. Figs. 1a, b; 2a–c; 3a–e; 4a–d, f; 6a–f).

In the zone between the entrance of the eye nerve into the brain and the first visual neuropil, the eye nerve splits into several eye nerve bundles again. The single bundles are surrounded by cell bodies. The single nerve bundles also have a ‘plaited‘ appearance. Again here we exclusively found axons, dendrites of interneurons and/or synaptic connections were absent and no genuine chiasmatic fibres were observed (Figs. 2b; 6a, d–g).

### Median eye visual neuropils

Proximal to the entrance of the eye nerve into the brain, we found a large neuropil complex extending from the tapered protrusion to the arcuate body (Figs. 1, 2, 3, 4). In the DiI/DiO staining experiments the neuropil complex appears as one single neuropil (Fig. 3a–d). Only in Fig. 3e the neuropil complex is subdivided into a brighter part with plenty dye and a darker part with fewer dye. With osmium ethyl gallate procedure the neuropil complex is also subdivided in a bright stained area distally and a dark stained area proximally (Fig. 4d, g). A somewhat different situation is found in the cobalt fills (Figs. 1b–g; 2a–c, f, g). The distal part (same as the bright stained area in the osmium ethyl gallate procedure) is densely filled with cobalt, followed by a thin transition zone and proximally a thick zone with few cobalt filled axons (same as the distal part of the dark stained area in the osmium ethyl gallate procedure).

**Fig. 7:**
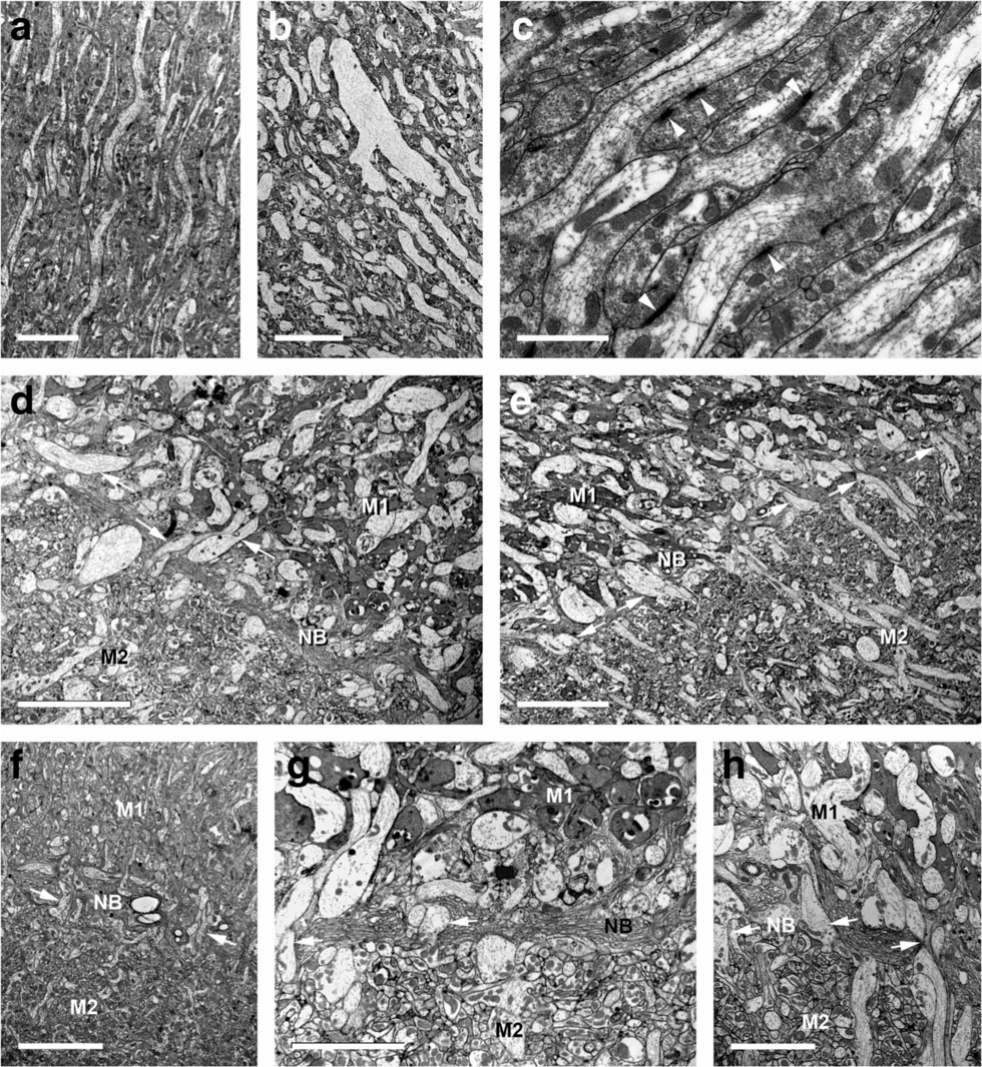
Transmission electron microscopy of first and second visual neuropil of *Platybunus pinetorum* (a, c, and f sagittal sections; b, d, e, g, and H transversal sections; dorsal is up). **a**, **b** showing arrangement of retinula axons (bright cells with high electron density) and dendrites of visual second order neurons (dark cells with low electron density) in first visual neuropil. Bars 5 μm and 10 μm, respectively. **c** detail of first visual neuropil with synapses (arrowheads) between retinula axons and visual second order neurons. Bar 2 μm. **d**, **e** transition area between first and second visual neuropil with neuropil border; note first and second neuropil with different anatomy and neuron gestalten; note several retinula axons traverse neuropil border (arrows). Bars 10 μm. **f**, **g** and **h** detail of transition area between first and second visual neuropil with various retinula axons traversing neuropil border (arrows). Bars 10 μm, 5 μm, and 5 μm, respectively. M, median eye visual neuropil; NB, neuropil border

In the following the distal part of the median eye neuropil complex is interpreted as the first median eye visual neuropil and the proximal part as the second median eye visual neuropil, separated by a neuropil border. That these regions certainly are neuropils is visible with TEM, where dendrites of interneurons and synaptic connections are visible (Fig. 7). Furthermore in particular TEM and osmium ethyl gallate procedure show that there are indeed two separate visual neuropils. The two neuropils have with both methods a different appearance. In TEM the first visual neuropil has large cell profiles and parallel fibres, the second mostly smaller cell profiles and, at first sight, a chaotic cell arrangement (Fig. 7d–h). In osmium ethyl gallate procedure the first neuropil is bright stained and the second dark stained (Fig. 4d, g). In addition the neuropils are separated by the neuropil border (see below).

### First median eye visual neuropil

The first median eye visual neuropil receives input from all R-cell axons from the visual nerve. The neuropil is pear-shaped and about 200 μm long and 100 μm wide. Within the neuropil the axons maintain their parallel orientation; this can be visualised with osmium ethyl gallate procedure (Fig. 4c, d) and especially TEM (Fig. 7a–c). The electron lucent R-cell axons contain numerous lateral protrusions, varicosities, and synaptic contacts indicating that this neuropil section is a first order neuropil. Between the axons are numerous arborisations of visual interneurons, giving this region its typical neuropil structure.

### Neuropil border

Proximal to the first neuropil, there is a transition zone of about 10 μm thickness (Figs. 1b–g; 4a–d, g; 7d-h). This zone is the neuropil border between the first and second visual neuropil. It contains numerous small, dark stained profiles of neurons and/or glia cells and thick, bright stained fibres, most probably R-cell axons. These represent only a portion of the visual fibres; that means that most of the R-cells terminate in the first neuropil and only few R-cells traverse the border and terminate in the second. Within the border no chiasmatic fibres were observed (Fig. 7d-h).

### Second median eye visual neuropil

Proximal to the neuropil border the second median eye visual neuropil begins. The neuropil is roundish with a diameter of about 100 μm. Cobalt fills show that only in the distal part of the second neuropil cobalt filled R-cells are found. These are the visual fibres seen in TEM that project through the neuropil border. They terminate in the first third of the neuropil. Hence, the second visual neuropil is subdivided in a part with direct R-cell input and a part without.

### Arcuate body

The arcuate body is located proximal to the second visual neuropil (Fig. 4e–h). These neuropils are in direct contact with each other (Fig. 4h). The arcuate body is horseshoe-shaped, slightly bent anteriorly and surrounded by a cell body rind.

## Discussion

**Fig. 8:**
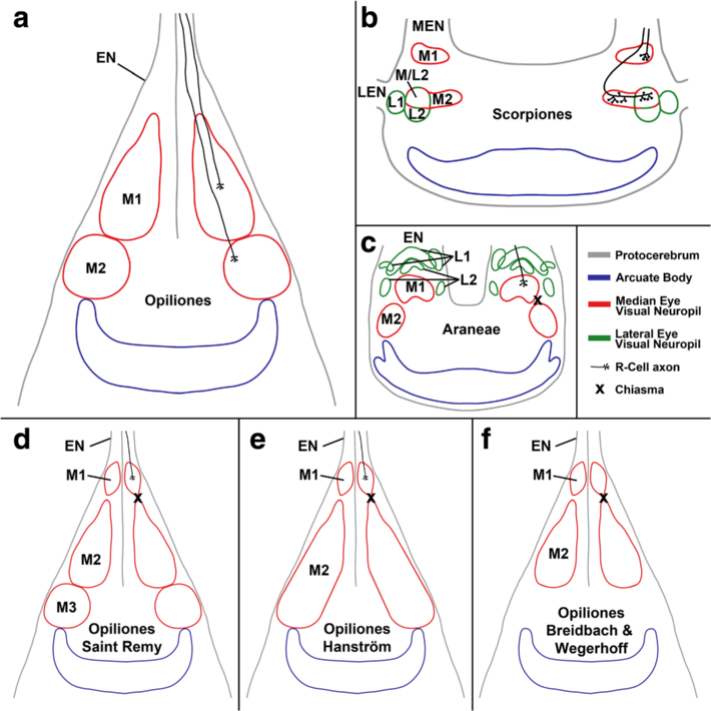
Comparison of the results of this study (a) with other taxa (b, c) and with previous studies (d–f). **a** Opiliones, this study, note region, that in previous studies (d–f) is described as M1, is indeed eye nerve and M1 lies deeper in the protocerebrum; **b** Scorpiones (*Euscorpius italicus*, *E. hadzii*; after Lehmann & Melzer [15]); **c** Araneae (*Cupiennius salei*; after Strausfeld et al. [22]); **d** Opiliones, after Saint Remy [25]; **e** Opiliones, after Hanström [29], note M2 subdivided into three layers (layer C, B, and C, see text) by Hanström; **f** Opiliones, after Breidbach & Wegerhoff [30], note M2 subdivided into two layers (internal and external, see text) by Breidbach & Wegerhoff. EN, eye nerve; L, lateral eye visual neuropil; LEN, lateral eye nerve; M, median eye visual neuropil; M/L2, region where M2 and L2 overlap; MEN, median eye nerve

Previous studies of the median eye visual neuropils of Opiliones were contradictory [25, 26, 29, 30]. The present re-examination comes to yet another conclusion concerning the number and position of the visual neuropils (see also Table 1 and Fig. 8).

**Table 1:** Comparison of the results of this study with the studies of Saint Remy, Holmgren, Hanström, and Breidbach & Wegerhoff [25–27, 29, 30]

This study	Saint Remy	Holmgren	Hanström	Breidbach & Wegerhoff
‘plaited’ nerve (without chiasma)	couche fibro-médullaire supérieure	three neuropils (“Sehmassen”), without mapping	first optic mass	first optic lobe
	couche des fibrilles chiasmatiques		chiasma	chiasma 1
first visual neuropil	couche fibro-médullaire inférieure		second optic mass, layer A	second optic lobe, external
neuropil border (without chiasma)	séparés par une substance plus claire (neuropil border)		second optic mass, layer B	second optic lobe, internal
second visual neuropil	masse médullaire		second optic mass, layer C	unnamed area and chiasma 2

Saint Remy [25] wrote a detailed work on the organization of arthropod brains, particularly those of harvestmen, and described the visual neuropils of the latter as highly developed. Beneath each eye he counted 7–8 axon bundles running to the brain ventrally. These bundles fuse to the two optic nerves along the way. This observation has been confirmed in the present study. Saint Remy's detailed description of the visual neuropils comprises a total of four layers. The first layer, or first visual neuropil, which he called “couche fibro-médullaire supérieure”, is perceived here not as a neuropil, but as an eye nerve (Fig. 6). Saint Remy described the following “couche des fibrilles chiasmatiques” as an elongated chiasma where the axons intersect at acute angles. This chiasma has not been found in the present study (Fig. 6). The third layer, or “couche fibro-médullaire inférieure”, corresponds to the first visual neuropil described by us. Saint Remy’s description of the region between the third and fourth layer as a neuropil border is in accordance with our findings. The fourth layer, or “masse médullaire”, corresponds to the second visual neuropil in the present study. Saint Remy's report included structures that we call axon terminals today in the first layer only. In contrast, we have detected such terminals in the first neuropil (Saint Remy's third layer), but some axons cross the neuropil border and terminate in the second visual neuropil (Saint Remy's fourth layer) (Fig. 7d–h).

Holmgren [26] largely confirmed Saint Remy's observations. He emphasised that there are three visual neuropils, unfortunately without giving a detailed description or illustration.

Hanström [29] reduced the number of visual neuropils to just two. His first “optic mass” with subsequent chiasma corresponds to the “couche fibro-médullaire supérieure” and “couche des fibrilles chiasmatiques” of Saint Remy, a region identified as a nerve in this study. Hanström’s second “optic mass” is subdivided in 3 layers (“a, b, and c”) and combines the “couche fibro-médullaire inférieure” (Saint Remy) or first visual neuropil (this study) as layer A, the neuropil border (both studies) as layer B, and the “mass médullaire” (Saint Remy) or second visual neuropil (this study) as layer C. According to Hanström these three layers are morphologically undivided and surrounded by a continuous layer of ganglion cells. The only differences he perceived between these layers concerned their stainability and the fibre pathways. This interpretation is not shared here; in contrast, the region of the protocerebrum is seen as two separated neuropils. Like Saint Remy, Hanström reported that all retinal fibres terminate in the first “optic mass” with slightly thickened ends. The staining experiments performed by us show that the fibres terminate in the first and second visual neuropils (layers A and C of Hanström's second optic mass).

Breidbach & Wegerhoff [30] gave an interpretation of the conditions in the visual system of harvestmen similar to Hanström's. They disagreed substantially, however, in seeing the second “optic lobe” as composed of only two layers (external and internal), whereas Hanström's layer C (equivalent to the second visual neuropil in the present study) was not mentioned by Breidbach & Wegerhoff. In this area they described a second chiasma only, a configuration that is not supported here. Furthermore, it was not specified where the R-cell axons terminate. Breidbach & Wegerhoff mentioned a columnar organisation of the external layer of the second “optic lobe”. In the present study the first visual neuropil – which corresponds to that external layer in position and form – is also described as columnar.

Comparing the results of the present study with those by Saint Remy, Hanström, and Breidbach & Wegerhoff [25, 26, 29, 30] one finds numerous discrepancies. This comparison is summarised in the Table 1 and in Fig. 8. The main difference concerns the first neuropil and the subsequent chiasma described by Saint Remy, Hanström, and Breidbach & Wegerhoff. In some of our preparations, especially in cobalt fills (e.g., Fig. 1d) this region looked neuropil-like, whereas in most other preparations (e.g., Figs. 1a; 4c, d, f) it looked nerve-like. However, TEM – from two different angles (transversal and sagittal) – clearly showed that this region represents a nerve. TEM allows an unequivocal distinction between nerve and neuropil. Accordingly, we found no synapses or dendrites of second order neurons, just axons (Fig. 6). No chiasma is evident, although the nerve does give a ‘plaited’ appearance. The latter probably misled the earlier authors to describe a chiasma. In this region, groups of axons are just interwoven with their neighbours. Hence, a primitive form of retinotopic projection arrangement of these nerve bundles occurs, resembling that in Pycnogonida [14]. All nerve fibres from the eye are bundled and a re-assortment of the single axons takes place. Consequently, the second visual neuropil of the three earlier studies (couche fibro-médullaire inférieure in Saint Remy, layer A in Hanström, internal layer in Breidbach & Wegerhoff) actually is the first median eye visual neuropil. Proximal to the first neuropil we found the same result as Saint Remy: a neuropil border and another median eye visual neuropil. Hanström and Breidbach & Wegerhoff saw this neuropil border as a neuropil-layer. Furthermore, the second median eye visual neuropil in our view is in Hanström's layer C. Breidbach & Wegerhoff did not mention this neuropil at all, but it is visible in their Fig. 7a, b.

To sum up these findings, the present re-examination analyses successfully the pathway of the R-cell axons in the visual system of several phalangid harvestmen species, and the construction of their visual neuropils. Just distal to the tapered area of entrance to the brain, the several eye nerve bundles from the eye join and form a single nerve per hemisphere. This nerve is composed of densely packed axons. Here, a retinotopic projection arrangement takes place. From each eye the R-cell axons supply two distinct, successive visual neuropils. The first median eye neuropil receives input from all R-cell axons from the visual nerve. It is located in the anterodorsal tip of the protocerebrum. The neuropil has a parallel or columnar orientation of the visual fibres, with large cell profiles. The second median eye neuropil lies proximally to the first. It is subdivided in a part with direct R-cell input and a part without. The two visual neuropils are separated by a neuropil border, with a part of the whole R-cell axons traversing the border. In TEM the second neuropil looks different from the first neuropil, with mostly smaller cell profiles and – at first sight – no special arrangement. Hence, this area of the protocerebrum is interpreted as two separate median eye neuropils rather than as a single neuropil. Subsequent to the second visual neuropil the arcuate body is found; both neuropils contact each other. No chiasma was found, neither before the first neuropil nor between the first and second neuropils. A summary of the basic features of the visual system in Opiliones is given in Fig. 8a.

## Conclusions

Phalangida have only one pair of median eyes, while the visual system of most other chelicerates consists of several pairs of eyes – median eyes and lateral eyes (e.g., Xiphosura, Scorpiones, Araneae, Uropygi, and Amblypygi). Besides Opiliones, the basal Pycnogonida and Solifugae possess only median eyes, and Pseudoscorpiones possess only lateral eyes. However, examined in detail are only the visual systems of Pycnogonida, Xiphosura (*Limulus*), Scorpiones, and Araneae [14–24, 31]. Concerning the R-cell projections and neuropil arrangement, two different configurations have been described, with Pycnogonida, Xiphosura, and Scorpiones on the one side and Araneae on the other.

The innervation pattern of the eyes in pycnogonids is similar to that of the median rudimentary eyes in *Limulus*. In both taxa the R-cell axons have collaterals in two target regions, in a first visual neuropil (or ocellar ganglion) and in a second visual neuropil near the arcuate body [14, 20]. In Scorpiones the cells of the median eye retina are also linked to two visual neuropils: the photoreceptor cells to a first visual neuropil, and the arhabdomeric cells to a second neuropil. The R‐cells of the lateral eyes are linked to a first and a second visual neuropil as well. Furthermore, the second median and the second lateral eye visual neuropils overlap each other; this means that there is a region with axon terminals from both eye types [15, 31]. A similar situation is found in the normal median and lateral eyes of Xiphosura [17, 18, 20], indicating close evolutionary relationships, at least of the visual systems. A chiasma in the median eye visual system is found neither in Pycnogonida, nor in Xiphosura, nor in Scorpiones. Finally – as in Opiliones – in the median eye retinae of *Limulus* and scorpions arhabdomeric cells are found.

In contrast, in Araneae the first anterior median eye neuropil is the only target region of R‐cells of the median eyes (principal eyes or anterior median eyes) [22]. It is located laterally in each brain hemisphere. Subsequent second‐order neurons terminate in a second visual neuropil (medulla). Between the first and second neuropils a chiasma is described. In addition, a tract that extends into the arcuate body has been suggested. However, only photoreceptor cells but no arhabdomeric cells are described from the retina of the studied spider species. Hence, a connection from these cells to the second visual neuropil – as in scorpions – is missing.

Lastly, in the visual system of Opiliones (Phalangida) an intermediate situation is observed. A comparison with Scorpiones and Araneae is shown in Fig. 8. As in Pycnogonida, Xiphosura, and Scorpiones the R-cell axons of the median eye have two target neuropils, a first and a second visual neuropil, but no chiasma is found. However, in Xiphosura (normal median eye) and Scorpiones the photoreceptor cells terminate in the first visual neuropil and the arhabdomeric cells in the second one. In the retina of phalangid Opiliones also photoreceptor and arhabdomeric cells are found, but proximal to the nuclear region within the eye nerve and the neuropil the two cell types are indistinguishable in TEM [10, 11]. For this reason a distinction of their respective target neuropils could not be made in this study. In contrast, the general arrangement of the neuropils involved in the visual system of harvestmen closely resembles that in Araneae. In both groups the second visual neuropil is directly adjacent to the first visual neuropil proximally, and to the arcuate body distally. In Xiphosura and Scorpiones these tree neuropils are in entirely different regions of the protocerebrum and do not contact each other. The first visual neuropil is located anterodorsally in the lateral part of the protocerebrum, whereas the second visual neuropil lies deeper in a more ventral and anterior position, and the arcuate body is found in a superficial dorso-posterior position.

It appears that the median eye visual neuropils of scorpions and *Limulus* represent the ancestral state and the median eyes of Araneae the derived state, with an intermediate situation in phalangid Opiliones. If in harvestmen – as in scorpions and *Limulus* – the photoreceptor cells project to the first and the arhabdomeric cells to the second visual neuropil, this would mean that harvestmen have spider visual neuropils with scorpion/*Limulus* projections.

Once more the analysis of the visual system in a chelicerate order has provided several characters for phylogenetic comparisons, but some questions remain unsolved. In order to characterise the ground pattern in all of Opiliones, the visual neuropils in the sister group of Phalangida, Cyphophthalmi, should be investigated in depth as well. The eyes of Cyphophthalmi have been discussed either as median eyes [8] or as lateral eyes [1]. Furthermore, the presence/absence of arhabdomeric cells and the targets of their projections need to be examined in detail. At this point it is far too early to draw phylogenetic conclusions on these observations, as too few arachnid orders have been studied; data are missing, for example, on Pseudoscorpiones or Solifugae.

## Methods

### Specimen collection

Specimens of *Leiobunum spec*., *Opilio canestrinii* (Thorell, 1876), *Platybunus pinetorum* (C. L. Koch, 1839), and *Rilaena triangularis* (Herbst, 1799) were collected in Munich between September and December 2013 and in April 2016.

### Cobalt fills

*Leiobunum spec*. and *Opilio canestrinii*, modified after Altman & Tyrer [32]: CoCl_2_ crystals were inserted in eyes with a fine tungsten needle. After diffusion times between 1 and 4 h, Cobalt was precipitated with a solution of five drops of (NH_4_)_2_S in 10 ml H_2_Odest. After fixation of the cephalothorax in AAF (85 ml 100% ethanol, 10 ml 37% formaldehyde, 5 ml glacial acetic acid), the specimen were silver intensified: 60 min at 50°C in dark in solution A (10 ml H_2_Odest, 3 ml 100% ethanol, 0.5 g gum arabic, and 0.02 g hydroquinone; pH value adjusted to between 2.6 and 3.1 using citric acid), and 15–30 min at 50°C in the dark in solution B (10 ml H_2_Odest, 3 ml 100% ethanol, 0.5 g gum arabic, 0.02 g hydroquinone, 0.01 g AgNO_3_; pH value adjusted to between 2.6 and 3.1 using citric acid). Silver intensification was stopped in an acetic acid solution (50 ml 30% ethanol, 5 g glucose, pH value adjusted to between 2.6 and 3.1 using acetic acid). After dehydration in a graded acetone series, the specimen were embedded in Glycidether 100, and sectioned with a rotary microtome and stainless steel blade in the sagittal, frontal, and transversal planes (14 μm). Some sections were silver intensified in solution A and B for a second time.

### DiI/DiO labelling

*Leiobunum spec*. and *Opilio canestrinii*, after Wohlfrom & Melzer [33]: The cephalothorax was dissected and fixed overnight at 4°C in 4% formaldehyde in 0.1 M PBS. Afterwards specimens were rinsed overnight in 0.1 M PBS, 0.1% NaN_3_. Finally, small DiI or DiO crystals (Molecular Probes) were inserted in eyes with a fine tungsten needle. Diffusion was carried out in darkness on small glass slides enclosed in wet chambers for 2–7 days. To prevent the growth of microorganisms, NaN_3_ in PBS was used for moistening. From time to time the specimens were controlled under the microscope. Specimens were studied with a fluorescence microscope and CLSM (LEICA DMRBE and Leica SP5).

### Osmium ethyl gallate procedure

*Leiobunum spec*., modified after Wigglesworth, Leise & Mulloney, and Mizunami et al. [34–36]: Specimen were dissected and fixed in 4% glutardialdehyde in 0.1 M cacodylate buffer at 4°C. After postfixation in 2% OsO_4_ in 0.1 M cacodylate buffer (3 h at 4°C) animals were stained for 17 h at 4°C in a saturated ethyl gallate solution, dehydrated in a graded acetone series, embedded in Glycidether 100, and sectioned with a rotary microtome and stainless steel blade in the sagittal and transversal planes (8 μm).

### TEM

*Rilaena triangularis*: After dissection the specimen were fixed in 4% glutardialdehyde in 0.1 M cacodylate buffer at 4°C. After postfixation in 2% OsO_4_ in 0.1 M cacodylate buffer (3 h at 4°C) the specimen were dehydrated in a graded acetone series and embedded in Glycidether 100. Ultra-thin sections of 70–100 nm thickness were made with a diamond knife on an RMC-MTXL ultramicrotome. The sections were stained with uranyl actetate and lead citrate, and inspected in an FEI Morgagni transmission EM at 80 kV.

### 3D-reconstruction

Specimen (prepared as for Osmium ethyl gallate procedure) was cut into a complete transversal series (8 μm). Slices were mounted on glass slides, covered with cover slips, and photographed under a conventional light microscope. Images were contrast-enhanced in Adobe Photoshop, then aligned, segmented and rendered in Amira.
